# COVID-19 Pandemic and Maternal Psychological Wellbeing During the Malaysian Movement Control Order: A Cross-Sectional Study

**DOI:** 10.3389/fpsyt.2021.745034

**Published:** 2022-01-04

**Authors:** Aida Kalok, Syarifah Aminah Syed Anwar Aly, Rahana Abdul Rahman, Zaleha Abdullah Mahdy, Shalisah Sharip

**Affiliations:** ^1^Obstetrics and Gynaecology Department, Faculty of Medicine, National University of Malaysia, Universiti Kebangsaan Malaysia Medical Centre, Kuala Lumpur, Malaysia; ^2^Psychiatry Department, Faculty of Medicine, National University of Malaysia, Universiti Kebangsaan Malaysia Medical Centre, Kuala Lumpur, Malaysia

**Keywords:** COVID-19, pregnancy, depression, anxiety, DASS-21, SWEMWBS

## Abstract

**Background:** COVID-19 pandemic had resulted in nationwide lockdown as a disease control measure. Potential harm to self and baby due to COVID-19 infection as well as uncertainties about delivery are among contributors to maternal anxiety. We aimed to assess the prevalence of psychological distress among pregnant women during the Malaysian Movement Control Order (MCO).

**Methods:** A cross-sectional study was conducted between May and June 2020 in a teaching hospital in Kuala Lumpur, Malaysia. A self-administered electronic questionnaire was distributed which included the following; (1) Depression, Anxiety and Stress Scale-21 (DASS 21), (2) Short Warwick Edinburgh Mental Wellbeing Scale (SWEMWBS), (3) MCO effect questionnaire, and (4) newly designed COVID-19 pregnancy-related anxiety. Chi-square test and logistic regression were performed to determine significant associations whilst mean scores comparison were conducted through Mann-Whitney-*U*-test.

**Results:** Four hundred and fifteen women were included in the final analysis. The prevalence of psychological distress among our cohort was 14.7%; with a two-fold increase of risk among the non-Malays (AOR 1.98, 95% CI 1.00–3.89) whilst a greater number of social support showed a protective effect (AOR 0.51, 95%CI 0.28–0.92). Malay ethnicity (*p* < 0.001) alongside greater household income (*p* = 0.014) were positive predictors of a higher sense of maternal wellbeing. Multiparous women and those of higher economic status experienced the more negative effect of the MCO. Around 88% of our women reported a higher level of COVID-19 pregnancy-related anxiety. Younger (*p* = 0.017) and first-time mothers (*p* = 0.039) were more likely to be anxious. Although adequate maternal knowledge on COVID-19 was associated with a greater sense of maternal wellbeing (*p* = 0.028), it was also linked to a higher level of COVID-19 related anxiety (AOR 3.54, 95% 1.29–9.70).

**Conclusion:** There was a relatively low prevalence of psychological distress among expectant mothers in Malaysia during the first wave of the COVID-19 pandemic. Expectant mothers should receive accurate and reliable information on the effect of COVID-19 on pregnancy to relieve some maternal anxiety. Maternal health screening is important to identify individuals who would benefit from extra support and mental health intervention, especially in prolonged lockdown.

## Introduction

Corona Virus Disease 2019 (COVID-19) is caused by the severe acute respiratory syndrome coronavirus 2 (SARS-CoV-2) which was first identified in Wuhan, China. The rapid spread of the virus since its first detection in December 2019, had resulted in a global outbreak and the declaration of a pandemic by the World Health Organization (WHO) on 11 March 2020. To date, around 188 million people were infected worldwide with four million recorded mortalities (1). Malaysia reported over nine hundred thousand cases with a death rate of 0.77% (2).

Public health measures such as accurate and early detection of SARS-CoV-2, combined with isolation and contract tracing of positive cases, are essential to prevent further community spread (3). Mass quarantine is also implemented by authorities in the effort to reduce COVID-19 transmission. Similar to other countries, the Malaysian Government had implemented nationwide lockdown in the form of movement control order (MCO). The MCO involved the closure of the international border as well as all educational and business premises (4). Any mass gathering was strictly prohibited and only selected providers of essential services such as food, health, telecommunication, and transportation were allowed to operate (5).

The risk of severe coronavirus disease 2019 (COVID-19) among pregnant women may be higher than in the general population. Physiological and immunity changes during pregnancy increase the women's susceptibility to severe disease (6). Data from the United States' Centre for Disease Control and Prevention (CDC) indicated that pregnancy was significantly associated with an increased chance of ICU admission, the need for invasive ventilation, and maternal mortality (7).

The combination of disease pandemic and national lockdown would inevitably lead to psychological distress and a low state of wellbeing among pregnant women (8). Psychological wellbeing is a mixture of pleasant emotion and the ability to function effectively in the personal and social domain (9). The nature of psychological wellbeing is multi-dimensional, which includes a sense of control, supportive social relations, and general satisfaction with life (10). Previous studies have demonstrated the negative correlation between psychological wellbeing and distress (5, 10). Labrague et al. found that higher levels of fear of COVID-19 were associated with increased psychological distress, lesser job satisfaction, reduced health perceptions and greater turnover intention (11).

Risk of COVID-19 transmission to self and fetus alongside uncertainties about deliveries, loss of household income, and domestic conflict was among the stressors which contributed toward pregnancy-related anxiety (12). Studies conducted during the first wave of the pandemic showed an increase in the prevalence of depression and anxiety among pregnant women (13–15). Wu et al. found that the depressive rates were positively associated with the number of newly-confirmed COVID-19 cases and fatalities (15). Primiparity, younger age, lack of social support, and previous psychiatric diagnosis were among the risk factors for maternal depression and anxiety (14).

Evidence shows that prenatal psychological distress affects both maternal and fetal wellbeing, with a potentially long-term effect on child development (16, 17). Maternal stress in pregnancy may lead to poor maternal psychosocial function and parenting difficulties, whilst adverse effects on the fetus include growth restriction, alteration in brain development, prematurity, and low birth weight (18). Recent evidence has demonstrated the negative impact of prenatal depression and anxiety on the socio-emotional (19) and cognitive development of the offspring (20); with an increased risk of depression in adolescence and adulthood (21). Therefore, in this era of the COVID-19 pandemic, maternal mental health wellbeing should be considered an important public health issue. We aimed to assess the prevalence of psychological distress among Malaysian pregnant women during the COVID-19 nationwide lockdown. Our study objectives also included the evaluation of maternal COVID-19 pregnancy-related anxiety and the impact of MCO on their psychological wellbeing.

## Materials and Methods

We conducted a cross-sectional study during the Malaysian MCO; from May 2020 till June 2020 among women who received obstetric care in a teaching hospital in Kuala Lumpur. Prior study approval was obtained from the Ethics Committee of Universiti Kebangsaan Malaysia (FF-2020-211). The inclusion criteria were pregnant Malaysian women aged above 18 years old and able to understand the Malay language, whilst exclusion criteria were women with abnormal fetuses or stillbirth. Participants recruitment was conducted among women who: (1) attended the outpatient clinic for an antenatal appointment, and (2) admitted to the obstetric ward for delivery or other medical complications. Eligible women were invited to complete the electronic version of the questionnaire through Google form, which contained a consent section. Socio-demographics and clinical data were included in the data collection. We also evaluated the maternal knowledge, perception, and practice during the COVID-19 pandemic as part of this research and the relevant results had already been published (22).

### Instruments

Our survey was conducted in Malay, the country's national language. The following instruments were used for data collection.

#### Depression, Anxiety and Stress Scale-21 and Psychological Distress

DASS 21 is a self-reporting tool measuring characteristic attitudes and symptoms of depression, anxiety, and stress (23). There are seven items for each emotional state. The Malay version of the questionnaires had been validated and demonstrated to have good psychometric properties for the general Malaysian population (24) with an overall Cronbach's alpha of 0.90 (25). The participants were asked to rate the extent to which they have experienced various symptoms over the past week, and the score for each subscale was calculated based on the previous study (26). A respondent who demonstrated symptoms of depression, anxiety, or stress from the calculated score would be considered as experiencing psychological distress.

#### Short Warwick Edinburgh Mental Wellbeing Scale

The Short Warwick–Edinburgh Mental Wellbeing Scale (SWEMWBS) is a shorter version of the 14-items Warwick–Edinburgh Mental Wellbeing Scale (WEMWBS), which was originally developed to monitor wellbeing in the general population (27), and to evaluate policies addressing wellbeing (27–29). There are seven positively worded items, each with five response categories (1, none of the time; 5, all of the time). The score range is 7–35, and higher scores indicate greater mental wellbeing (30). The Malay version of the SWEMWBS had been validated in a Malaysian cohort and was negatively correlated with depression, anxiety, and stress (5). The mean score for SWEMWBS was used to determine the threshold for a higher sense of wellbeing among our cohort.

#### Perception of the Effect of MCO on Self Wellbeing

We assessed the maternal perception of the effect of MCO on their wellbeing using a three-item questionnaire which was developed by Kalok et al. The questionnaire previously demonstrated a good internal consistency (Cronbach's alpha 0.86) (5). The participants were asked to rate each statement using a scale; 1 (strongly disagree) to 7 (strongly agree):

- MCO has disrupted your daily life.
- MCO has affected your physical wellbeing.
- MCO has affected your mental/psychological wellbeing.

The total score for all three responses was calculated, and a cut-off level based on the mean score was determined (31, 32). A total score above this level would indicate that the MCO was perceived to have a negative effect on the woman's wellbeing.

#### COVID-19 Pregnancy-Related Anxiety

A five-item questionnaire was designed to assess COVID-19 pregnancy-related anxiety. Each woman was asked to respond to each item on COVID-19 using a scale; 1 (not worried at all) to 7 (very worried):

- Infection to self
- Infection to baby
- COVID-19 causing miscarriage
- COVID-19 causing preterm birth
- COVID-19 causing abnormality to baby

The total score ranged from 7 to 35. We used a 50% cut-off level (score ≥ 18) to indicate greater maternal anxiety.

#### Social Support

We had asked the participants to indicate their perceived source of social support. Social support may be in various forms including emotional, physical, or even financial. The participants were given a list that includes family, friends, employer, and government; and they were free to choose as many as they deemed relevant.

#### COVID-19 Related Knowledge

We developed a nine-item questionnaire that covered the sign and symptoms of COVID-19, methods of transmission, and disease prevention; based on the available literature. Total knowledge score ranged from 0 to 9. Participants' overall knowledge was categorized as adequate if the score was more than 50% (22). The association between maternal knowledge and psychological distress, maternal wellbeing, perceived MCO effect, and COVID-19 related anxiety was evaluated in this study.

### Statistical Analysis

This study was part of research that included the evaluation of maternal knowledge on COVID-19. Our sample size was determined based on the assumption that the probability of having adequate maternal knowledge was 50.0% (33). Taking into consideration, 95% confidence interval, the limit of precision 5%, with a design effect of 1.0, the calculated sample size was 384 participants.

The study data were analyzed using the Statistical Package of Social Sciences (SPSS) Version 24.0 (IBM Corp., Armonk, NY, USA). Data were presented as mean (standard deviation, SD) or number, n (percentage, %) for continuous and categorical data, respectively. The scores for DASS 21, SWEMWBS, MCO effect, and COVID-19 pregnancy-related anxiety were inspected for normality using the Kolmogorov–Smirnov test. The internal consistency of all the questionnaires was assessed using Cronbach's alpha. Cronbach's alpha > 0.7 was regarded as satisfactory. All of the items in the newly designed COVID-19 related anxiety questionnaire underwent exploratory factor analysis to confirm the number of factors. The Kaiser rule (Eigenvalue > 1.0) was applied to determine the number of dimensions to extract, whilst the sampling adequacy was assessed through Bartlett's test of sphericity and Kaiser–Meyer–Olkin (KMO). The correlations between the COVID-19 pregnancy-related anxiety and psychological distress, maternal wellbeing, and MCO effect were assessed using Pearson's correlation.

Chi-square test and univariate analysis were performed to determine the significant factors associated with (1) maternal psychological distress, (2) higher sense of maternal wellbeing, (3) negative effect of MCO, and (4) greater COVID-19 pregnancy-related anxiety. The statistically significant variables were analyzed in the multiple variable logistic regression, which included the adjustment for age, ethnicity, and parity, to produce the adjusted odd ratios (AORs) and the corresponding 95% confidence interval. The mean scores for SWEMWBS, MCO effect, and COVID-19 related anxiety were also compared using different maternal characteristics. A *p*-value <0.05 was considered statistically significant.

## Results

We approached four-hundred and fifty women for this study and 93% completed the questionnaire. Five women were excluded due to incomplete data; resulting in a total number for analysis of 415. The demographic and clinical characteristics of our cohort are demonstrated in Table 1. The mean (SD) age for our women was 32.4 (4.5) and the majority (85%) were Malays. Over three-quarters of our women received tertiary education and were employed. Around 60% of participants were pregnant whilst the remaining was post-partum. The mean (SD) gestation was 31.8 (8.3) weeks. Almost two-fifths of our women had medical or obstetric complications. Table 1 also depicts the social support received by the women. The majority of women received family support (92%). The proportions who reported employer and government support were 49 and 61%, respectively. Our study also found that around 95% of our participants demonstrated adequate knowledge of COVID-19.

**Table 1:** Demographics, clinical characteristics, social support, and maternal knowledge.

**Maternal characteristics**	***n* (%)**
Age, mean (SD)	32.4 (4.5)
**Ethnicity**, ***n*** **(%)**	
Malay	353 (85.1)
Chinese	40 (9.6)
Indian	11 (2.7)
Others	11 (2.7)
**Education**, ***n*** **(%)**	
Primary	2 (0.5)
Secondary	94 (22.7)
Tertiary	319 (76.9)
**Employment**, ***n*** **(%)**	
Employed/self employed	322 (77.6)
Housewife	91 (21.9)
Student	2 (0.5)
**Household income**	
<RM 2,000	25 (6.0)
RM 2,000–4,999	178 (42.9)
RM 5,000–10,000	177 (42.7)
>RM 10,000	35 (8.4)
Critical sector employment	157 (37.8)
Parity, mean (SD)	1.7 (1.3)
Nulliparous	68 (16.4)
Multiparous	347 (83.6)
Antenatal	240 (57.8)
Postnatal	175 (42.2)
Vaginal birth	107 (25.8)
Cesarean delivery	68 (16.4)
Gestation, mean (SD)	31.8 (8.3)
**Medical or Obstetrics complication**, ***n*** **(%)**	157 (37.8)
Hypertensive disorder	26 (6.3)
Diabetes	89 (21.4)
Anemia	17 (4.1)
Others	25 (6.0)
**Social support**, ***n*** **(%)**	
Family	383 (92.0)
Friends	234 (56.4)
Employer	204 (49.2)
Government	256 (61.7)
**COVID-19 related knowledge**	
Inadequate	21 (5.1)
Adequate	394 (94.9)

*SD, standard deviation; RM, Ringgit Malaysia; COVID-19, Corona Virus Disease 2019*.

### Psychological Distress

The Cronbach alpha for the Malay version of DASS-21 was 0.960. The proportion of our women who reported symptoms of depression, anxiety, and stress were 4.3, 14.0, and 5.8%, respectively; as demonstrated in Table 2. The prevalence of psychological distress in our cohort was 14.7% as sixty-one women scored positive in at least one category. We found that the non-Malays were almost twice more likely to report symptoms of depression, anxiety, or stress (*p* = 0.049) whilst those with a greater number of social support were almost 50% less likely to suffer from psychological distress (*p* = 0.026); as demonstrated in Table 3.

**Table 2:** Depression, anxiety, and stress prevalence according to categories.

**DASS-21 category**	**Depression** ***n* (%)**	**Anxiety** ***n* (%)**	**Stress** ***n* (%)**
Normal	397 (95.7)	357 (86.0)	391 (94.2)
Mild	8 (1.9)	36 (8.7)	23 (5.5)
Moderate	8 (1.9)	14 (3.4)	1 (0.3)
Severe	2 (0.5)	7 (1.7)	–
Extremely severe	–	1 (0.2)	–

*DASS-21, Depression, Anxiety and Stress Scale 21-item*.

**Table 3:** Relationship between maternal demographics and psychological distress.

**Factors**	**Psychological distress**	
	***n* (%)**	**AOR (95% CI)**
**Age**		
<35	35 (13.5)	Ref
>35	26 (16.7)	1.18 (0.68–2.07)
	*p* = 0.380	*p* = 0.555
**Ethnicity**		
Malay	47 (13.3)	Ref
Non-Malay	14 (22.6)	1.98 (1.00–3.89)
	*p* = 0.057	*p* = 0.049
**Income**		
<RM 5,000	30 (14.8)	Ref
>RM 5,000	31 (14.6)	0.95 (0.55–1.66)
	*p* = 0.964	*p* = 0.861
**Education**		
Non-tertiary	13 (13.5)	Ref
Tertiary	48 (15.0)	1.22 (0.62–2.39)
	*p* = 0.715	*p* = 0.560
**Employment**		
Employed/self-employed	43 (13.4)	Ref
Others	18 (19.4)	1.46 (0.79–2.71)
	*p* = 0.150	*p* = 0.228
**Parity**		
Nulliparous	6 (8.8)	Ref
Multiparous	55 (15.9)	2.02 (0.82–4.95)
	*p* = 0.135	*p* = 0.126
**Medical condition**		
Yes	34 (13.2)	Ref
No	27 (17.2)	1.33 (0.76–2.34)
	*p* = 0.262	*p* = 0.320
**Source of social support**		
1–2	39 (19.5)	Ref
3 or more	22 (10.2)	0.51 (0.28–0.92)
	*p* = 0.008	*p* = 0.026
**COVID-19 Knowledge**		
Inadequate	5 (23.8)	Ref
Adequate	56 (14.2)	0.62 (0.21–1.80)
	*p* = 0.226	*p* = 0.377

*AOR, adjusted odd ratio; CI, confidence interval; Ref, reference; COVID-19, Corona Virus Disease 2019. AOR was adjusted for age, ethnicity, and parity*.

### Short Warwick Edinburgh Mental Wellbeing Scale

The mean (SD) SWEMWBS score among our cohort was 26.90 (6.40). The seven-item questionnaire demonstrated good internal consistency with Cronbach's alpha of 0.961. SWEMWBS was positively correlated with depression, stress, and anxiety (*p* < 0.001) as depicted in Table 4. Table 5 demonstrates the comparison of mean scores among women with different characteristics. The data analysis was performed through the Mann-Whitney-*U*-test as the SWEMBBS scores were not normally distributed. We found that the Malay women (*p* = 0.001) and those with greater income (*p* = 0.033) displayed significantly higher wellbeing scores. We used the total score of 26 and above as an indicator of a higher sense of maternal wellbeing. Chi-square and logistic regression analysis confirmed that Malay ethnicity along with greater household income (>RM5000) as an independent predictor of a higher sense of maternal wellbeing as shown in Table 6. Adequate maternal knowledge was positively associated with a greater sense of maternal wellbeing; however, the association was not significant after adjustment for age, ethnicity, and parity.

**Table 4:** Pearson's correlations of DASS 21 domains, SWEMWBS, MCO effect, and COVID-19 anxiety.

	**Depression**	**Anxiety**	**Stress**	**SWEMWBS**	**MCO effect**
Depression	1				
Anxiety	0.822	1			
	*p* < 0.001				
Stress	0.880	0.882	1		
	*p* < 0.001	*p* < 0.001			
SWEMWBS	−0.211	−0.172	−0.196	1	
	*p* < 0.001	*p* < 0.001	*p* < 0.001		
MCO Effect	0.093	0.095	0.099	−0.168	1
	*p* = 0.057	*p* = 0.052	*p* = 0.045	*p* = 0.001	
COVID-19 anxiety	−0.088	−0.051	−0.051	0.185	0.009
	*p* = 0.075	*p* = 0.303	*p* = 0.301	*p* < 0.001	*p* = 0.856

*SWEMWBS, Short Warwick–Edinburgh Mental Wellbeing; MCO, Movement Control Order*.

**Table 5:** Comparison of maternal scores based on different maternal characteristics^*^.

**Maternal characteristics**	**Mean (SD)**		
	**SWEMWBS**	**MCO effect**	**COVID-19 pregnancy-related anxiety**
All, mean (SD)	26.90 (6.40)	8.66 (5.40)	28.73 (7.91)
**Age**			
<35	26.73 (6.82)	8.64 (5.51)	29.10 (7.42)
≥35	27.18 (5.63)	8.71 (5.25)	28.13 (8.67)
	*p* = 0.905	*p* = 0.660	*p* = 0.546
**Ethnicity**			
Malay	27.33 (6.20)	8.46 (5.42)	29.28 (7.57)
Non-Malay	24.42 (6.98)	9.84 (5.19)	25.65 (9.12)
	*p* = 0.001	*p* = 0.028	*p* = 0.001
**Education**			
Non-tertiary	26.14 (6.52)	7.30 (4.92)	30.09 (7.97)
Tertiary	27.13 (6.35)	9.07 (5.48)	28.33 (7.86)
	*p* = 0.159	*p* = 0.002	*p* = 0.011
**Employment**			
Employed/self-employed	26.83 (6.37)	8.81 (5.47)	28.65 (7.74)
Others	27.13 (6.52)	8.14 (5.15)	29.04 (8.52)
	*p* = 0.328	*p* = 0.246	*p* = 0.313
**Household income**			
<RM 5,000	26.38 (6.24)	7.58 (4.92)	28.86 (8.00)
≥RM 5,000	27.39 (6.52)	9.70 (5.65)	28.62 (7.85)
	*p* = 0.033	*p* < 0.001	*p* = 0.662
**Parity**			
Nulliparous	27.94 (5.74)	7.35 (4.55)	29.99 (6.02)
Multiparous	26.69 (6.51)	8.92 (5.53)	28.49 (8.22)
	*p* = 0.231	*p* = 0.038	*p* = 0.344
**Knowledge on COVID-19**			
Inadequate	24.52 (8.50)	8.14 (5.40)	23.76 (9.70)
Adequate	27.02 (6.26)	8.69 (5.41)	29.00 (7.73)
	*p* = 0.334	*p* = 0.549	*p* = 0.005
**Source of social support**			
1–2	26.50 (6.42)	8.72 (5.31)	28.23 (8.32)
3 or more	27.27 (6.37)	8.61 (5.50)	29.21 (7.51)
	*p* = 0.251	*p* = 0.840	*p* = 0.186

*SD, standard deviation; SWEMWBS, Short Warwick–Edinburgh Mental Wellbeing; MCO, Movement Control Order*.

* *Mann-Whitney test*.

**Table 6:** Associations between maternal characteristics and maternal wellbeing, MCO effect & COVID-19 pregnancy-related anxiety.

**Maternal characteristics**	**Higher maternal wellbeing**		**Negative MCO effect**		**Greater COVID-19 anxiety**	
	*n* (%)^*^	**AOR (95%CI)**	*n* (%)^*^	**AOR (95%CI)**	*n* (%)^*^	**AOR (95%CI)**
**Age**						
<35	174 (67.2)	Ref	122 (47.1)	Ref	236 (91.1)	1.86 (1.01–3.43)
≥35	113 (72.4)	1.40 (0.89–2.20)	72(46.2)	0.92 (0.61–1.37)	130 (83.3)	Ref
	*p* = 0.262	*p* = 0.141	*p* = 0.851	*p* = 0.668	*p* = 0.017	*p* = 0.046
**Ethnicity**						
Non-Malay	31 (50.0)	Ref	35 (56.5)	Ref	49 (79.0)	Ref
Malay	256 (72.5)	2.80 (1.61–4.90)	159 (45.0)	0.61 (0.35–1.06)	317 (89.8)	2.48 (1.21–5.08)
	*p* < 0.001	*p* < 0.001	*p* = 0.097	*p* = 0.080	*p* = 0.015	*p* = 0.013
**Education**						
Non-tertiary	59 (61.5)	Ref	35 (36.5)	Ref	87 (90.6)	Ref
Tertiary	228 (71.5)	1.47 (0.90–2.40)	159 (49.8)	1.87 (1.15–3.01)	279 (87.5)	0.66 (0.30–1.43)
	*p* = 0.062	*p* = 0.126	*p* = 0.021	*p* = 0.011	*p* = 0.400	*p* = 0.290
**Employment**						
Employed/self-employed	221 (68.6)	Ref	152 (47.2)	Ref	283 (87.9)	Ref
Others	66 (71.0)	1.26 (0.75–2.13)	42 (45.2)	0.86 (0.54–1.38)	83 (89.2)	1.25 (0.59–2.67)
	*p* = 0.668	*p* = 0.383	*p* = 0.728	*p* = 0.541	*p* = 0.721	*p* = 0.559
**Household income**						
<RM 5,000	128 (63.1)	Ref	77 (37.9)	Ref	177 (87.2)	Ref
≥RM 5,000	159 (75.0)	1.73 (1.12–2.67)	117(55.2)	2.10 (1.41–3.15)	189 (89.2)	1.35 (0.73–2.50)
	*p* = 0.008	*p* = 0.014	*p* < 0.001	*p* < 0.001	*p* = 0.536	*p* = 0.343
**Parity**						
Nulliparous	50 (73.5)	Ref	28 (41.2)	Ref	65 (95.6)	Ref
Multiparous	237 (68.3)	0.67 (0.37–1.23)	166 (47.8)	1.38 (0.81–2.36)	301 (86.7)	0.30 (0.09–1.01)
	*p* = 0.393	*p* = 0.200	*p* = 0.314	*p* = 0.240	*p* = 0.039	*p* = 0.051
**Knowledge on COVID-19**						
Inadequate	10 (47.6)	Ref	10 (47.6)	Ref	14 (66.7)	Ref
Adequate	277 (70.3)	2.32 (0.94–5.76)	184 (46.7)	1.05 (0.43–2.57)	352 (89.3)	3.54 (1.29–9.70)
	*p* = 0.028	*p* = 0.069	*p* = 0.934	*p* = 0.908	*p* = 0.002	*p* = 0.014
**Source of social support**						
1-2	132 (66.0)	Ref	95 (47.5)	Ref	173 (86.5)	Ref
3 or more	155 (72.1)	1.05 (0.67–1.64)	99 (46.0)	1.06 (0.71–1.58)	193 (89.8)	1.17 (0.61–2.23)
	*p* = 0.179	*p* = 0.834	*p* = 0.767	*p* = 0.790	*p* = 0.303	*p* = 0.644

*AOR, adjusted odd ratio; CI, confidence interval; Ref, reference; MCO, movement Control Order; COVID-19, Corona Virus Disease 2019*.

*AOR was adjusted for age, ethnicity, and parity*.

* *Chi-square test*.

### Perception of the Effect of MCO on Self Wellbeing

Table 7 demonstrates the perceived effect of MCO on maternal wellbeing. Almost 30% of our women found that the MCO disrupts their daily lives. Around one-fifth of them reported that the lockdown had a negative effect on their physical and emotional wellbeing. Our study found a positive correlation between the MCO effect score and stress (*p* = 0.045); as shown in Table 4, whilst a greater MCO effect score is associated with a lesser maternal sense of wellbeing (*p* = 0.001).

**Table 7:** Perceived effect of MCO on maternal wellbeing.

**Negative effect of MCO**	**Disagree**	**Neutral**	**Agree *n* (%)**
Disruption to the daily routine	233 (56.1)	61 (17.4)	121 (29.2)
Negative effect on physical wellbeing	293 (70.6)	31 (7.5)	91 (21.9)
Negative effect on emotional wellbeing	298 (71.8)	32 (7.7)	85 (20.5)

*MCO, Movement Control Order*.

We found that women who were non-Malay, tertiary educated, those with greater household income, and multiparous displayed significantly higher scores (Table 5); suggesting the unfavorable effect of the lockdown. The mean (SD) score for the MCO effect was 8.66 (5.40). Women who scored 8 and above were considered to have experienced the negative effect of MCO. Multivariable logistic regression confirmed tertiary education and greater household income as positive predictors of the negative effect of MCO lockdown among our cohort (Table 6).

### COVID-19 Pregnancy-Related Anxiety

Table 8 depicts the maternal response toward COVID-19 pregnancy-related worry. Over four-fifths of mothers worry about COVID-19 infection to themselves and their babies. Around 70% of our women had a concern about COVID-19 causing miscarriage, fetal abnormality, and preterm birth.

**Table 8:** COVID-19 pregnancy-related anxiety.

**Psychological effect**	**Disagree** ***n* (%)**	**Neutral** ***n* (%)**	**Agree** ***n* (%)**
**COVID-19 infection**			
Self	51 (12.3)	15 (3.6)	349 (84.1)
Unborn child/newborn	51 (12.3)	16 (3.9)	348 (83.9)
**COVID-19 effect on pregnancy**			
Miscarriage	71 (17.1)	50 (12.0)	294 (70.8)
Abnormal baby	69 (16.6)	50 (12.0)	296 (71.3)
Preterm delivery	71 (17.1)	44 (10.6)	300 (72.3)

*COVID-19, Corona Virus Disease 2019*.

The Cronbach's alpha for this questionnaire was 0.928. The Kaiser-Meyer-Olkin (KMO) was 0.776, and Bartlett's Test of Sphericity reached statistical significance with *p* < 0.001, supporting the sample factorability. Exploratory factor analysis on the five items confirmed single factor loading. Mann-Whitney-*U*-test revealed that Malay ethnicity, non-tertiary education, and adequate knowledge on COVID-19 were associated with higher anxiety scores (Table 5).

By using a fifty-percent threshold; we found that 88.2% of our women demonstrated a greater level of maternal anxiety. Age below 35, Malay ethnicity, and adequate knowledge of COVID-19 were associated with a greater level of COVID-19 pregnancy-related anxiety (Table 6).

## Discussion

The COVID-19 outbreak and nationwide lockdown had a significant psychological impact on vulnerable groups such as pregnant women. We presented the first study which evaluated the effect of the global pandemic on maternal mental health among Malaysian women. Our results demonstrated that Malay women had a lesser odd of experiencing psychological distress and were more likely to report a greater sense of maternal wellbeing. Women of higher socioeconomic status reported a higher sense of maternal wellbeing; however, they were also more likely to experience the negative effect of the lockdown. We also found that there was a high level of COVID-19 pregnancy-related anxiety among our women especially among the young and Malay mothers; as well as those who had sufficient knowledge on COVID-19.

Various studies on maternal mental health during the global pandemic showed an uptrend in the symptoms of psychological distress. A recent systematic review by Suwalska et al. found that the prevalence of depression and anxiety among perinatal women were as high as 56.3 and 77%, respectively (14, 34, 35). Current evidence also showed that the rate of psychological distress was positively correlated to the reported number of positive COVID-19 cases and related deaths (15, 36). This could explain the relatively low prevalence of depression and anxiety among our women, as this study was conducted in the early part of the COVID-19 pandemic in Malaysia, during which the country had just over eight thousand confirmed cases with only one-hundred twenty mortalities.

The COVID-19 outbreak had inevitably changed the delivery of health services including obstetric care. Hospitals had to ensure adequate resources to fight the pandemic by canceling elective procedures and outpatient clinics. Alteration to antenatal appointments (12) as well as uncertainty and concerns about perinatal care (37) were among the contributory factors of maternal depressive and anxiety symptoms. The absence of a partner during childbirth was also associated with a higher level of maternal anxiety (38). Several studies including ours have demonstrated the role of social support in reducing the risk of depression and anxiety among expectant mothers (37, 39, 40). Social support is essential in buffering the effect of stress in pregnancy as well as promoting maternal physical and psychological wellbeing (41). Women with a greater number of social support in our study demonstrated higher wellbeing scores, although the finding was not statistically significant.

In keeping with the previous study, we found a significant negative correlation between the SWEMWBS score and all the components of DASS 21 (3). Malay women and those of higher economic status reported a greater sense of maternal wellbeing in this study. Berthelot et al. demonstrated that pregnant women with low income were more prone to elevated distress and psychiatric symptoms during the COVID-19 pandemic (13). A Study conducted among Japanese women also found an increased odd of postnatal depression among those from the lower-income group (OR, 1.43; 95% CI: 1.03–1.98) (42). Although there was no significant difference in the psychological distress among women of different income brackets in our cohort, higher household income contributed to greater financial security to expectant mothers and resulted in an increased sense of wellbeing.

Nationwide lockdown had a significant socio-economic impact on the population. Closure of offices and business premises resulted in job losses and financial insecurity, whilst reduced family contact and restricted recreation activities would increase emotional stress.

Our study demonstrated that the lockdown has a negative effect on maternal wellbeing. We found that women with higher education and income level experienced a greater negative effect of MCO. These expectant mothers with higher economic status were more likely to have to work from home and found their social activities restricted during the lockdown. Multiparous mothers also demonstrated higher MCO effect scores compared to first-time mothers. Lack of childcare and online classes for school-going children might pose extra pressure on these mothers during the pandemic.

Over four-fifths of our women expressed worry about the risk of COVID-19 infection to themselves and their babies. Lebel et al. found that elevated symptoms of depression symptoms were associated with an increment in maternal concern on the COVID-19 threat to own life and potential harm to the baby (37). A study among pregnant individuals in Singapore during the early wave of the pandemic showed that women who associated COVID-19 infection with fetal anomalies and intrauterine fetal death had significantly higher anxiety scores (43). A study among Italian pregnant women during the COVID-19 national lockdown demonstrated that prenatal attachment negatively correlates with maternal state anxiety and depression. However, adequate and functional perception of COVID-19 could enhance prenatal attachment (44). The maternal-fetal emotional attachment is an important indicator of their health and the mother's efficiency in the postnatal period (45).

Currently, observational data have not suggested any link between COVID-19 and miscarriage, fetal anomaly, or spontaneous preterm births. Vertical transmission is plausible; however, the mechanisms are unclear and severe neonatal COVID-19 disease is fortunately rare (6). It is therefore important to fill the knowledge gap on the effect of COVID-19 on pregnancy by providing accurate and reliable information to expectant mothers. The younger and nulliparous women would benefit from extra support and reassurance as these groups exhibited a greater level of anxiety; in keeping with other studies (15, 42).

Our study evaluated the effect of adequate COVID-19 knowledge on maternal psychology during the pandemic. Liu et al. demonstrated that pregnant women with relatively more knowledge on COVID-19 and rational risk perception (not too nervous about epidemic control or going out) were less likely to be anxious (46). We found that individuals with adequate knowledge were more likely to report a greater sense of maternal wellbeing (OR 2.60, 95% CI 1.07–6.30, *p* = 0.034), however, the relationship was not significant following adjustment for age, ethnicity, and parity. These women also reported a higher level of COVID-19 pregnancy-related anxiety; which can be explained by the lack of available data on the effect of COVID-19 on pregnancy during the early wave of the pandemic.

Women's cultural background, which is influenced by race and religion; often plays a role in maternal perception, practice, and attitude toward the COVID-19 pandemic. Our previous published findings demonstrated that Malay women demonstrated a more positive attitude toward the MCO (22). A study from our neighboring country Singapore showed that pregnant Malay women were more likely to practice safe distancing and frequent hand sanitizing compared to the Chinese (47). The Malay women in our cohort were less likely to suffer from psychological distress. They also reported a greater sense of maternal wellbeing and experienced lesser negative effects from the lockdown. Good underlying knowledge on COVID-19 as well as high confidence in authority and health professionals among these women may explain the current findings (22).

### Strengths and Limitations

We presented the first Malaysian study that evaluated the psychological impact of COVID-19 on pregnant women during the first wave of the pandemic. The sample size was adequate to meet the statistical requirement and our newly designed COVID-19 pregnancy-related anxiety demonstrated good internal consistency and construct validity. Our study was among the few which assessed the influence of adequate knowledge of COVID-19 on maternal mental health. The validated Malay version of SWEMWBS is a valuable tool to evaluate the perception of wellbeing not only among pregnant women but also applicable to the general Malaysian population.

Our study is limited by its cross-sectional design. The data collection was conducted in the early part of the COVID-19 pandemic when there was little knowledge on the novel virus. This could explain the high level of COVID-19 pregnancy-related anxiety among our cohort. Unfortunately, the country is still under lockdown with an increased number of positive cases and deaths. Malaysia is suffering from the third wave of the pandemic and the health system is currently under strain from the exponential rise of severe cases (48). The prolonged social restriction will inevitably cause adverse effects on the population's mental health. A longitudinal study is therefore essential to assess the long-term impact of the pandemic and extended lockdown.

### Conclusion

The COVID-19 pandemic and national lockdown had a negative impact on maternal mental health wellbeing. Social support offers protection against psychological distress, as previously demonstrated by other studies. Dissemination of accurate information on COVID-19 effect on pregnancy is essential to address the knowledge gap among expectant mothers and reduce pregnancy-related anxiety. Mental health screening is essential in prolonged pandemics and lockdown to identify vulnerable individuals, in the effort to safeguard maternal psychological wellbeing and promote healthy prenatal attachment during this testing time.

## Data Availability Statement

The original contributions presented in the study are included in the article/supplementary material, further inquiries can be directed to the corresponding author/s.

## Ethics Statement

The studies involving human participants were reviewed and approved by Universiti Kebangsaan Malaysia Ethics Committee. The patients/participants provided their written informed consent to participate in this study.

## Author Contributions

AK, RA, and ZM: conceptualization. AK, SAS, and SS: methodology. AK and SS: formal analysis. AK and SAS: investigation. AK: writing—original draft preparation and project administration. AK, RA, ZM, and SS: writing—review and editing. RA: supervision. All authors have read and agreed to the published version of the manuscript.

## Conflict of Interest

The authors declare that the research was conducted in the absence of any commercial or financial relationships that could be construed as a potential conflict of interest.

## Publisher's Note

All claims expressed in this article are solely those of the authors and do not necessarily represent those of their affiliated organizations, or those of the publisher, the editors and the reviewers. Any product that may be evaluated in this article, or claim that may be made by its manufacturer, is not guaranteed or endorsed by the publisher.

## References

[1] World Health Organization. WHO Coronavirus(COVID-19) Dashboard. WHO (2021). Available from: https://covid19.who.int/ (accessed May 19, 2021).

[2] Malaysia MoH. Situasi Terkini COVID-19 di Malaysia [Latest update on COVID-19 in Malaysia]. Kuala Lumpur (2021). Available from: http://covid-19.moh.gov.my/terkini/2021/05/situasi-terkini-covid-19-di-malaysia (accessed May 19, 2021).

[3] Eftekhari A Alipour M Chodari L Maleki Dizaj S Ardalan M Samiei M. A comprehensive review of detection methods for SARS-CoV-2. Microorganisms. (2021) 9:1–19. 10.3390/microorganisms9020232 33499379 PMC7911200

[4] Singh J Singh J. COVID-19 and its impact on society. Electron Res J Soc Sci Human. (2020) 2:103–6.

[5] Kalok A Sharip S Abdul Hafizz AM Zainuddin ZM Shafiee MN. The psychological impact of movement restriction during the COVID-19 outbreak on clinical undergraduates: a cross-sectional study. Int J Environ Res Public Health. (2020) 17:1–13. 10.3390/ijerph17228522 33212969 PMC7698578

[6] Wastnedge EAN Reynolds RM van Boeckel SR Stock SJ Denison FC Maybin JA. Pregnancy and COVID-19. Physiol Rev. (2021) 101:303–18. 10.1152/physrev.00024.2020 32969772 PMC7686875

[7] Zambrano LD Ellington S Strid P Galang RR Oduyebo T Tong VT. Update: characteristics of symptomatic women of reproductive age with laboratory-confirmed SARS-CoV-2 infection by pregnancy status - United States, January 22-October 3, 2020. MMWR Morb Mortal Wkly Rep. (2020) 69:1641–7. 10.15585/mmwr.mm6944e3 33151921 PMC7643892

[8] Mortazavi F Mehrabadi M KiaeeTabar R. Pregnant women's well-being and worry during the COVID-19 pandemic: a cross-sectional study. BMC Pregn Childbirth. (2021) 21:59. 10.1186/s12884-021-03548-4 33451292 PMC7809640

[9] Ryff CD Singer B. Psychological well-being: meaning, measurement, and implications for psychotherapy research. Psychother Psychosom. (1996) 65:14–23. 10.1159/000289026 8838692

[10] Winefield HR Gill TK Taylor AW Pilkington RM. Psychological well-being and psychological distress: is it necessary to measure both? Psychol Well Being Theory Res Pract. (2012) 2:1–14. 10.1186/2211-1522-2-3

[11] Labrague LJ de Los Santos JAA. Fear of COVID-19, psychological distress, work satisfaction and turnover intention among frontline nurses. J Nurs Manag. (2021) 29:395–403. 10.1111/jonm.13168 32985046 PMC7537256

[12] Moyer CA Compton SD Kaselitz E Muzik M. Pregnancy-related anxiety during COVID-19: a nationwide survey of 2740 pregnant women. Arch Womens Ment Health. (2020) 23:757–65. 10.1007/s00737-020-01073-5 32989598 PMC7522009

[13] Berthelot N Lemieux R Garon-Bissonnette J Drouin-Maziade C Martel É Maziade M. Uptrend in distress and psychiatric symptomatology in pregnant women during the coronavirus disease 2019 pandemic. Acta Obstet Gynecol Scand. (2020) 99:848–55. 10.1111/aogs.13925 32449178

[14] Suwalska J Napierała M Bogdański P Łojko D Wszołek K Suchowiak S. Perinatal mental health during COVID-19 pandemic: an integrative review and implications for clinical practice. J Clin Med. (2021) 10:1–16. 10.3390/jcm10112406 34072357 PMC8199229

[15] Wu Y Zhang C Liu H Duan C Li C Fan J. Perinatal depressive and anxiety symptoms of pregnant women during the coronavirus disease 2019 outbreak in China. Am J Obstet Gynecol. (2020) 223:240.e1-.e9. 10.1016/j.ajog.2020.05.009 32437665 PMC7211756

[16] Laplante DP Barr RG Brunet A Galbaud du Fort G Meaney ML Saucier JF. Stress during pregnancy affects general intellectual and language functioning in human toddlers. Pediatr Res. (2004) 56:400–10. 10.1203/01.PDR.0000136281.34035.44 15240860

[17] Meaney MJ. Perinatal maternal depressive symptoms as an issue for population health. Am J Psychiatry. (2018) 175:1084–93. 10.1176/appi.ajp.2018.17091031 30068258

[18] Field T Diego M Hernandez-Reif M Figueiredo B Deeds O Ascencio A. Comorbid depression and anxiety effects on pregnancy and neonatal outcome. Infant Behav Dev. (2010) 33:23–9. 10.1016/j.infbeh.2009.10.004 19945170 PMC2819543

[19] Madigan S Oatley H Racine N Fearon RMP Schumacher L Akbari E. A meta-analysis of maternal prenatal depression and anxiety on child socioemotional development. J Am Acad Child Adolesc Psychiatry. (2018) 57:645–57.e8. 10.1016/j.jaac.2018.06.012 30196868

[20] Tarabulsy GM Pearson J Vaillancourt-Morel MP Bussières EL Madigan S Lemelin JP. Meta-analytic findings of the relation between maternal prenatal stress and anxiety and child cognitive outcome. J Dev Behav Pediatr. (2014) 35:38–43. 10.1097/DBP.0000000000000003 24345757

[21] Tirumalaraju V Suchting R Evans J Goetzl L Refuerzo J Neumann A. Risk of depression in the adolescent and adult offspring of mothers with perinatal depression: a systematic review and meta-analysis. JAMA Netw Open. (2020) 3:e208783. 10.1001/jamanetworkopen.2020.8783 32602910 PMC7327545

[22] Syed Anwar Aly SA Abdul Rahman R Sharip S Shah SA Abdullah Mahdy Z Kalok A. Pregnancy and COVID-19 pandemic perception in Malaysia: a cross-sectional study. Int J Environ Res Public Health. (2021) 18:1–11. 10.3390/ijerph18115762 34072017 PMC8198971

[23] Lovibond SH LP. Manual for the Depression Anxiety Stress Scales. 2nd ed. Sydney: Psychology Foundation of Australia (1995).

[24] Musa R Fadzil MA Zain Z. Translation, validation and psychometric properties of Bahasa Malaysia version of the Depression Anxiety and Stress Scales (DASS). ASEAN J Psychiatry. (2007) 8:82–9.

[25] Nordin RB Kaur A Soni T Por LK Miranda S. Construct validity and internal consistency reliability of the Malay version of the 21-item depression anxiety stress scale (Malay-DASS-21) among male outpatient clinic attendees in Johor. J Med J Malaysia. (2017) 72:265. 29197880

[26] Shamsuddin K Fadzil F Ismail WS Shah SA Omar K Muhammad NA. Correlates of depression, anxiety and stress among Malaysian university students. Asian J Psychiatr. (2013) 6:318–23. 10.1016/j.ajp.2013.01.014 23810140

[27] Stewart-Brown S Tennant A Tennant R Platt S Parkinson J Weich S. Internal construct validity of the Warwick-Edinburgh mental well-being scale (WEMWBS): a Rasch analysis using data from the Scottish health education population survey. Health Qual Life Outcomes. (2009) 7:15. 10.1186/1477-7525-7-15 19228398 PMC2669062

[28] Tennant R Hiller L Fishwick R Platt S Joseph S Weich S. The Warwick-Edinburgh Mental Well-being Scale (WEMWBS): development and UK validation. Health Qual Life Outcomes. (2007) 5:63. 10.1186/1477-7525-5-63 18042300 PMC2222612

[29] Cilar L Pajnkihar M Štiglic G. Validation of the Warwick-Edinburgh mental well-being scale among nursing students in Slovenia. J Nurs Manage. (2020) 28:1335–46. 10.1111/jonm.13087 32654293

[30] Stewart-Brown S Janmohamed K. Warwick-Edinburgh mental well-being scale. User guide Version. (2008) 1. 10.1037/t80221-000

[31] Arumugam B Suganya E Nagalingam S. Derivation of cut-off value for a 10 item opinion based ordinal survey questionnaire. Int J Commun Med Public Health. (2018) 5:1030. 10.18203/2394-6040.ijcmph20180756

[32] Field A. Discovering statistics using IBM SPSS statistics: sage (2013).

[33] Lemeshow S Hosmer DW Klar J Lwanga SK World Health Organization. Adequacy of Sample Size in Health Studies. Chichester: Wiley (1990).

[34] Mappa I Distefano FA Rizzo G. Effects of coronavirus 19 pandemic on maternal anxiety during pregnancy: a prospectic observational study. J Perinat Med. (2020) 48:545–50. 10.1515/jpm-2020-0182 32598320

[35] Kahyaoglu Sut H Kucukkaya B. Anxiety, depression, and related factors in pregnant women during the COVID-19 pandemic in Turkey: a web-based cross-sectional study. Perspect Psychiatr Care. (2021) 57:860–8. 10.1111/ppc.12627 32989798 PMC7537279

[36] Kotabagi P Fortune L Essien S Nauta M Yoong W. Anxiety and depression levels among pregnant women with COVID-19. Acta Obstet Gynecol Scand. (2020) 99:953–4. 10.1111/aogs.13928 32474914 PMC7300632

[37] Lebel C MacKinnon A Bagshawe M Tomfohr-Madsen L Giesbrecht G. Elevated depression and anxiety symptoms among pregnant individuals during the COVID-19 pandemic. J Affect Disord. (2020) 277:5–13. 10.1016/j.jad.2020.07.126 32777604 PMC7395614

[38] Molgora S Accordini M. Motherhood in the time of coronavirus: the impact of the pandemic emergency on expectant and postpartum women's psychological well-being. Front Psychol. (2020) 11:567155. 10.3389/fpsyg.2020.567155 33192847 PMC7649390

[39] Yue C Liu C Wang J Zhang M Wu H Li C. Association between social support and anxiety among pregnant women in the third trimester during the coronavirus disease 2019 (COVID-19) epidemic in Qingdao, China: the mediating effect of risk perception. Int J Soc Psychiatry. (2021) 67:120–7. 10.1177/0020764020941567 32643510 PMC7348553

[40] Taubman-Ben-Ari O Chasson M Abu-Sharkia S. Childbirth anxieties in the shadow of COVID-19: self-compassion and social support among Jewish and Arab pregnant women in Israel. Health Soc Care Community. (2020) 10.1111/hsc.13196 33058395 PMC7675716

[41] Dunkel Schetter C. Psychological science on pregnancy: stress processes, biopsychosocial models, and emerging research issues. Annu Rev Psychol. (2011) 62:531–58. 10.1146/annurev.psych.031809.130727 21126184

[42] Matsushima M Horiguchi H. The COVID-19 pandemic and mental well-being of pregnant women in Japan: need for economic and social policy interventions. Disaster Med Public Health Prep. (2020). 10.1017/dmp.2020.334. [Epub ahead of print]. 32907687 PMC7642494

[43] Ng QJ Koh KM Tagore S Mathur M. Perception and feelings of antenatal women during COVID-19 pandemic: a cross-sectional survey. Ann Acad Med Singap. (2020) 49:543–52. 10.47102/annals-acadmedsg.2020295 33164024

[44] Craig F Gioia MC Muggeo V Cajiao J Aloi A Martino I. Effects of maternal psychological distress and perception of COVID-19 on prenatal attachment in a large sample of Italian pregnant women. J Affect Disord. (2021) 295:665–72. 10.1016/j.jad.2021.08.102 34509782 PMC8428478

[45] Alhusen JL. A literature update on maternal-fetal attachment. J Obstet Gynecol Neonatal Nurs. (2008) 37:315–28. 10.1111/j.1552-6909.2008.00241.x 18507602 PMC3027206

[46] Liu X Chen M Wang Y Sun L Zhang J Shi Y. Prenatal anxiety and obstetric decisions among pregnant women in Wuhan and Chongqing during the COVID-19 outbreak: a cross-sectional study. Bjog. (2020) 127:1229–40. 10.1111/1471-0528.16381 32583536 PMC7362035

[47] Lee RWK Loy SL Yang L Chan JKY Tan LK. Attitudes and precaution practices towards COVID-19 among pregnant women in Singapore: a cross-sectional survey. BMC Pregn Childbirth. (2020) 20:675. 10.1186/s12884-020-03378-w 33167918 PMC7652671

[48] Dawn Chan ND. *Health DG: Malaysia in* 'very critical condition. Kuala Lumpur: New Staits Times (2021). Available from: https://www.nst.com.my/news/nation/2021/07/708003/health-dg-malaysia-very-critical-condition (accessed July 13, 2021).

